# A diffusion-based 3D printing strategy to fabricate self-supporting, perfusable networks

**DOI:** 10.1186/s44330-026-00059-6

**Published:** 2026-03-09

**Authors:** Daniel Ramos Mejia, Betty Cai, Sean Chryz Iranzo, Andy Perez, Yee Lin Tan, Seungheon Lee, Sarah C. Heilshorn

**Affiliations:** Department of Materials Science and Engineering, Stanford University, Stanford, CA 94305 USA

**Keywords:** Bioprinting, Biofabrication, Vascular mimics, Perfusable structures

## Abstract

**Background:**

Engineered vasculature is essential for the biofabrication of functional tissue mimics. To fabricate engineered vasculature, three-dimensional (3D) bioprinting has emerged as a promising approach due to its ability to form perfusable structures with customized geometries. Sacrificial ink extrusion, where sacrificial inks are printed into a crosslinkable hydrogel precursor support bath, is a versatile bioprinting modality for fabricating interconnected perfusable networks. However, the fabrication of self-supporting structures with a vessel-like shell remains challenging using conventional sacrificial ink extrusion approaches. To enable the fabrication of self-supporting, perfusable networks, we developed a 3D bioprinting approach termed Gelation of Uniform Interfacial Diffusant in Embedded 3D Printing (GUIDE-3DP). This approach leverages the diffusion of crosslinking initiators from a printed sacrificial ink into a gel precursor support bath to generate branched, perfusable networks with precise control over channel inner and outer diameters.

**Methods:**

Here, we present an end-to-end protocol for fabricating self-supporting vascular-like networks using the GUIDE-3DP method. We describe methods for freeform print path design, support bath and sacrificial ink preparation, 3D printing of perfusable structures, and seeding of printed structures with endothelial cells. Through this protocol, perfusable structures with complex branching geometries can be designed, fabricated, and endothelialized.

**Discussion:**

To highlight the ability of GUIDE-3DP to fabricate self-supporting, perfusable networks with complex geometries, we demonstrate the fabrication of three representative structures: (1) an interconnected retinal vasculature network, (2) a hierarchical branched vascular network, and (3) a dual-material capillary-like network. We further demonstrate the endothelialization of printed structures with one or two cell types via single- or dual-material printing. Beyond vascular-like networks, this protocol is readily adaptable to design and fabricate mimics of other perfusable structures in the human body.

**Clinical trial number:**

Not applicable.

**Supplementary Information:**

The online version contains supplementary material available at 10.1186/s44330-026-00059-6.

## Background

Vasculature is necessary for sustaining the viability of cells, tissues, and organs by facilitating the transport of oxygen, signaling molecules, nutrients and waste. Functional vascular networks are crucial for maintaining homeostasis and supporting cellular metabolism in both engineered and natural tissues. Without effective vascularization, engineered tissues would lack complexity, size, and long-term functionality. Despite this significance, the fabrication of complex, perfusable vascular networks has remained a crucial challenge in the field of tissue engineering and regenerative medicine [1].

To enable precise spatial control for fabricating tissue-like structures, 3D bioprinting has emerged over the past several decades as a promising technology with capabilities of precisely positioning biomaterials, cells, and growth factors [2, 3]. Many innovative bioprinting techniques have been developed to improve structural fidelity and spatial control for biofabrication of complex vascular networks [4–8]. Embedded 3D printing involves extruding bioinks within a support bath, improving the print fidelity of soft biomaterials and enabling the fabrication of non-planar geometries [9–11]. Layer-by-layer printing sequentially builds structures by stacking thin layers, allowing for precise architectural control [12–14]. Coaxial printing utilizes concentric nozzles to directly extrude hollow filaments, enabling the formation of perfusable tubular structures [15–18]. Sacrificial ink printing involves depositing a removable material within a bulk matrix to create interconnected channels or voids, which can later be cleared to form perfusable networks with smooth surfaces [19–21].

While Fickian diffusion principles have long been a foundational concept, their application to 3D bioprinting has gained significant attention as an emerging area of innovation [22–24]. In the context of bioprinting, crosslinking occurs when a diffusing crosslink initiator reaches a critical concentration threshold within a printed construct, triggering localized gelation. Accurate prediction and control of this process requires understanding time-dependent diffusion behavior, typically described by Fick’s laws. Experimental techniques such as fluorescence imaging and fluorescence recovery after photobleaching (FRAP) are commonly used to assess the diffusivity of fluorescent model molecules in bioinks and support materials [9, 22, 25, 26]. Computational modeling, particularly finite element modeling (FEM), plays a complementary role by simulating concentration gradients and crosslinking dynamics in complex geometries, enabling fine-tuned control over the structure and properties of bioprinted constructs [27, 28]. When combined with novel, diffusion-enabled bioprinting strategies, these methods allow for precise spatial patterning and tailored mechanical properties in bioprinted constructs, moving the field toward more predictive and programmable biofabrication.

To fabricate intricate and self-supporting vascular networks, we previously developed a bioprinting approach termed Gelation of Uniform Interfacial Diffusant in Embedded 3D Printing (GUIDE-3DP) [29]. This versatile technique leverages the diffusion of a crosslink initiator from a sacrificial ink to enable the creation of hollow, self-supporting and interconnected branched channels that mimic native vascular geometries. GUIDE-3DP uniquely enables the formation of self-supporting perfusable structures, addressing limitations of previous sacrificial ink extrusion methods. The GUIDE-3DP strategy is compatible with a variety of different crosslinking mechanisms to form perfusable channels with cell-compatible materials. These channels can be endothelialized post-printing through perfusion of a cell suspension, or directly during printing using a cell-laden sacrificial ink, allowing for seamless integration of vascular lining [30]. Compared to other embedded 3D bioprinting strategies, GUIDE-3DP combines the advantages of high spatial resolution, channel fidelity, and material versatility for fabricating self-supporting, hollow structures.

**Fig. 1:**
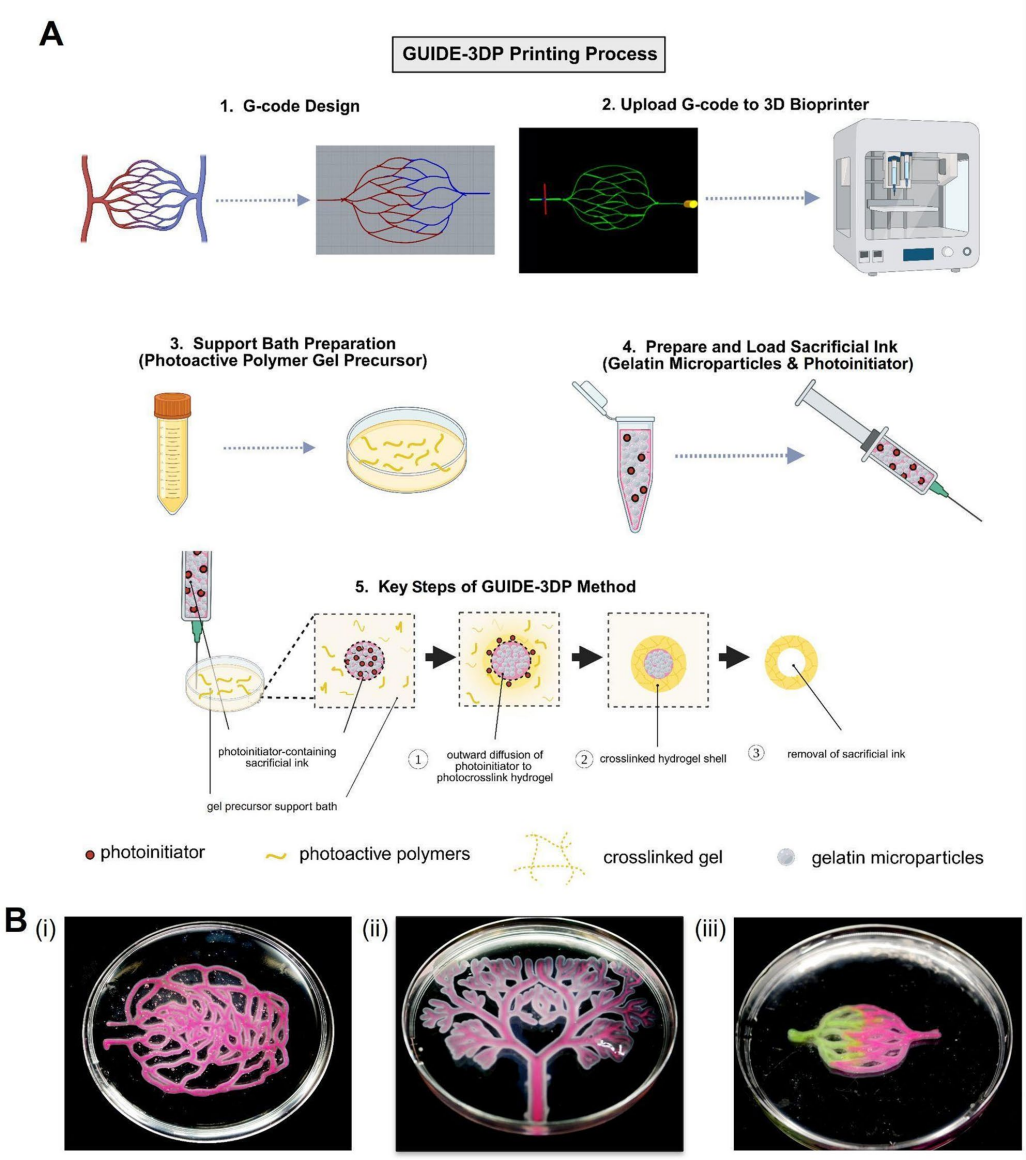
Gelation of Uniform Interfacial Diffusant in Embedded 3D Printing (GUIDE-3DP) approach for fabricating self-supporting, perfusable structures. **(A)** Key steps of the GUIDE-3DP process. **(B)** Representative vascular-like structures fabricated using GUIDE-3DP, positioned in a 10-cm dish: (i) retinal vasculature network (derived from the NIH3D repository [31]), (ii) branched vascular network (inspired by BioRender), and (iii) dual-material capillary-like network (inspired by BioRender)

Here, we describe the protocol for designing and fabricating branched vascular-like networks using the GUIDE-3DP technique and gelatin methacryloyl (GelMA). First, we present a workflow for designing freeform printing paths and converting them into G-codes for 3D printing. We then describe the end-to-end workflow for material preparation and print fabrication in GUIDE-3DP (Fig. 1A) for three representative vascular-like structures (Fig. 1B). We demonstrate control over the lumen diameter and vessel wall thickness by varying printing parameters such as the extrusion rate and crosslink initiator diffusion time. Beyond vascular applications, this method holds potential for generating other perfusable tissue models such as intestinal epithelia, fallopian tube analogs, and airway channels.

## Materials and procedure

The protocol described in this peer-reviewed article is published on protocols.io, updated July 26, 2025, 10.17504/protocols.io.5qpvowjx9l4o/v1. Additional details critical to the experimental procedures described are provided below.

### Modeling and g-code design

#### Vascular-like print path design

**Fig. 2:**
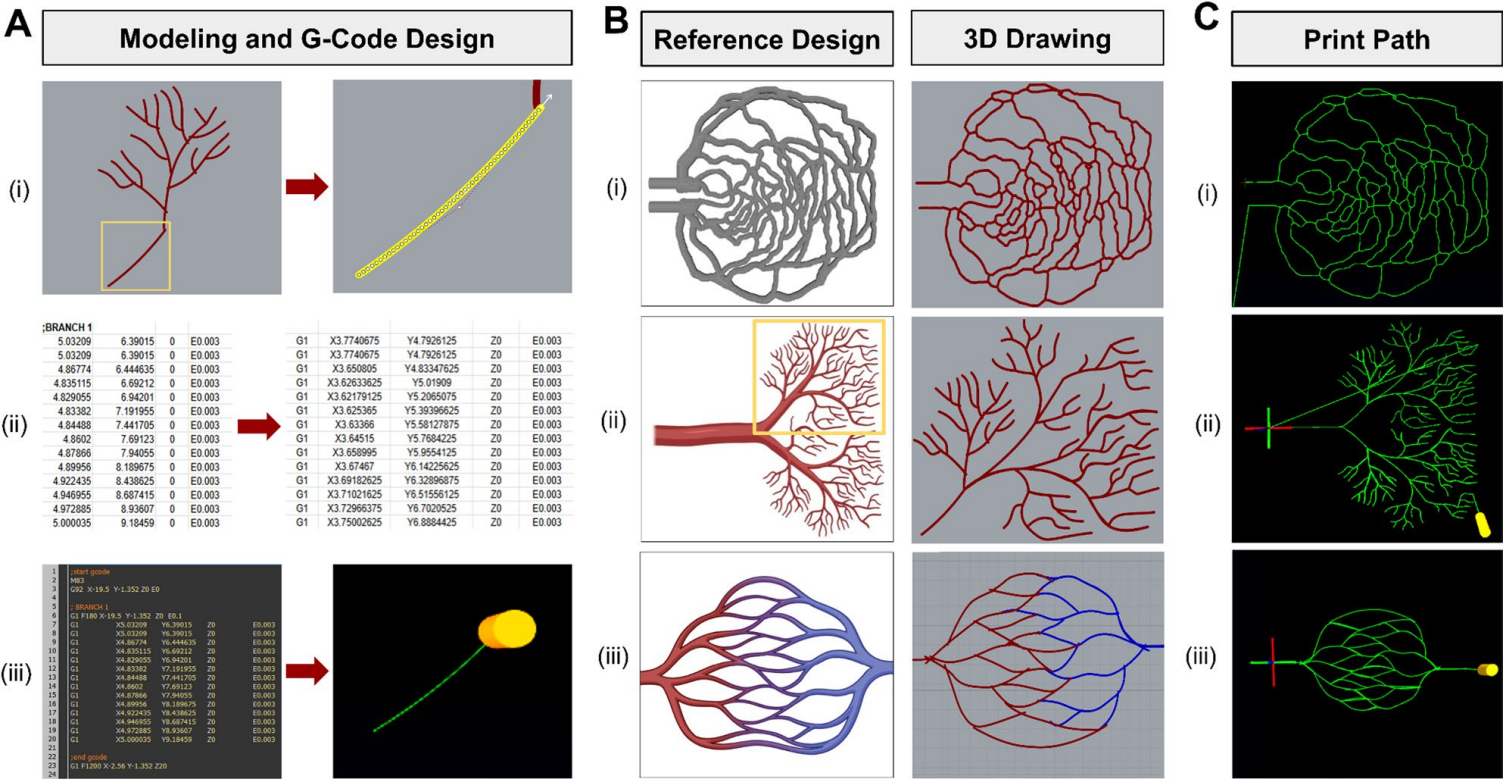
Modeling of vascular-like networks. **(A)** Key steps of modeling and G-code design process: (i) Sketch the first section of the structure, use the ‘Divide’ command on the first line, and verify the direction of printing (indicated as a white arrow); (ii) convert coordinates in the .txt file to G-code syntax via Excel; and (iii) copy G-code into editing software and verify its corresponding print path. **(B)** Drawings of three representative structures: (i) retinal vasculature network (derived from NIH3D [31]), (ii) branched vascular network (inspired by BioRender), and (iii) dual-material capillary-like network (inspired by BioRender). **(C)** Visualization of G-code print paths for the three representative structures inspired by panel B

The design of complex vascular-like geometries for 3D printing can be conducted using 3D modeling software. Here, we demonstrate the modeling of print paths using Rhinoceros 3D. Before beginning, users may optionally select reference images or models from publicly available databases such as NIH3D, BioRender, and the Vascular Model Repository [32]. To initiate the design of the structure, the “Control Point Curve” feature may be used along with other features to fulfill architectural needs. When drawing these lines, a slight overlap for each individual print path may be designed to prevent any gaps in the inner lumen during printing. Once the vascular network has been completed (Fig. 2A), each individual segment is extracted by the following steps: (1) selecting the starting segment of the print path, (2) using the “Divide” feature to divide the path into evenly spaced points, and (3) exporting the point coordinates into a .txt file (Fig. 2B).

The filament diameter is determined by a combination of the point spacing interval and the extrusion value. With a greater spacing between points in the print path or a lower extrusion value, the amount of ink being extruded will be reduced, resulting in a smaller filament diameter. For a point spacing interval of 0.5 mm, extrusion values (E) ranging from 0.005 to 0.02 mm of extrusion movement are typically appropriate.

#### Arrangement of G-code

The resultant print path is visualized using G-code editing software such as CAMotics to verify print size and direction (Fig. 2C). The .txt file with XYZ coordinates is input into Excel for conversion into G-code syntax, which follows the format “G1 X(#) Y(#) Z(#) E(#)” (Additional File 1). An example formula for the conversion from point coordinates to G-code syntax is “=IF(ISBLANK(B2),NA(),“X"&B2)”, where X can be interchangeable with Y and Z. Once all coordinates are converted into the new G-code syntax, it can be copied and pasted into a selected G-code editing environment. To enable printing, add commands prior to G-code start: define relative extrusion mode “M83”, then establish the starting coordinates in the format “G92 X(#) Y(#) Z(#) E0” (Additional File 2). A print speed (F value), defined in millimeters per minute, should be specified in the first line of the print path G-code.

The conversion from point coordinates to G-code is repeated for all segments of the print path. It is recommended to simulate each segment’s G-code individually to verify path direction and accuracy. In some cases, reversing the direction of a segment may be necessary and can be accomplished using Excel’s sorting function. To prevent unintended extrusion during movement between non-contiguous segments, it is preferable to re-trace along the printed path in reverse, with extrusion set to zero, to return to a continuing component rather than tracing the nozzle directly across empty space, which risks dragging the sacrificial ink to an unwanted area. A vertical lift movement may be appended at the end of the file to retract the nozzle above the support bath, thus minimizing disturbance of the printed geometry.

### Synthesis of gelatin methacryloyl (GelMA)

#### Support bath selection for GUIDE-3DP

The use of hydrogel support baths has become essential in the field of 3D bioprinting, particularly for fabricating freeform structures such as vascular-like networks. Selecting the ideal biomaterial to create the support bath is critical. The support bath must be shear thinning to allow the print nozzle to smoothly translate and have the necessary mechanical self-healing properties to provide sufficient support for temporarily holding the structure during printing, thus allowing stable, high-fidelity structures. Additionally, it should be biocompatible, easy to remove, and capable of mixing with other additives to improve its rheological properties. The GUIDE-3DP approach can be used with a wide range of support baths, including photocrosslinkable materials such as GelMA, polyethylene glycol diacrylate (PEGDA), and hyaluronic acid methacrylate (HAMA); enzymatically crosslinkable materials such as gelatin and fibrin; and ionically crosslinkable materials such as alginate and polyacrylamide [29]. In our demonstration, GelMA was selected due to its biocompatibility, excellent mechanical properties, and ability to be crosslinked on demand. Raising the GelMA concentration increases the density of polymer chains and crosslinking sites, which consequently elevates the hydrogel modulus. GelMA concentrations of 10–20 wt% are typically appropriate for the generation of perfusable structures in GUIDE-3DP [29, 30].

Here, we describe a protocol for GelMA synthesis enabling the large-scale preparation of gel precursor support baths for GUIDE-3DP.

#### GelMA reaction

First, GelMA from cold water fish (CWF) skin-derived gelatin is synthesized using a protocol suitable for large reaction scales (e.g. 60 g of gelatin in 300 mL). Unlike porcine gelatin, CWF gelatin remains in liquid form at room temperature, making it suitable for use as a support bath in GUIDE-3DP. GelMA is prepared by reacting gelatin with methacrylic anhydride through the primary amines on lysine residues.

**Fig. 3:**
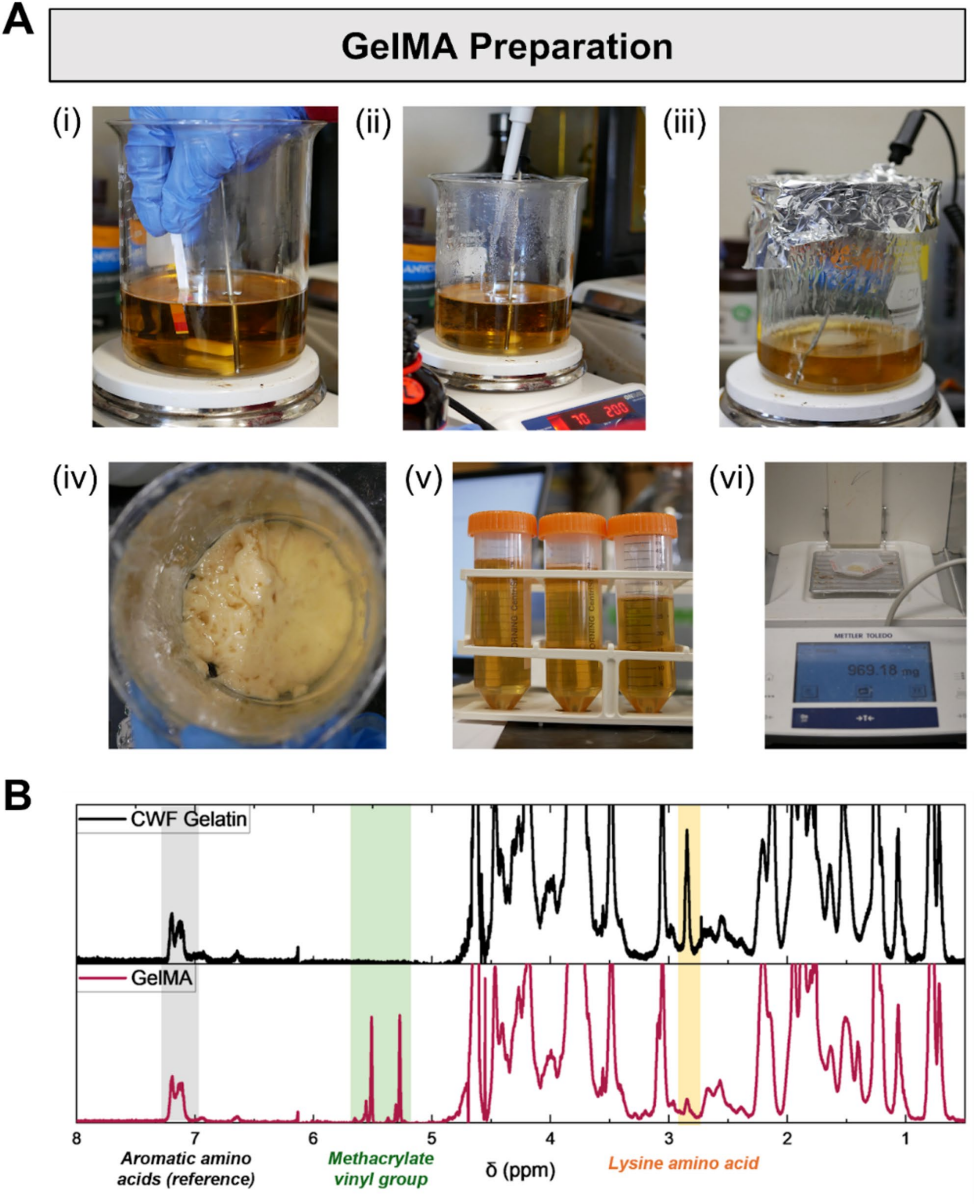
Preparation of cold water fish (CWF) GelMA. **(A)** Key steps of GelMA preparation process: (i) pH adjustment of gelatin solution; (ii) addition of MAA to gelatin solution; (iii) reaction of gelatin with MAA in a covered beaker at 70℃; (iv) aggregated GelMA after ethanol precipitation of the reaction mixture; (v) GelMA stock solution after redissolution and residual ethanol removal; and (vi) measurement of GelMA stock concentration. **(B)** Representative ^1^H NMR spectra of CWF gelatin and GelMA purified by dialysis

The process starts with the preparation of a gelatin solution (20% w/v) in 1 M carbonate-bicarbonate (CB) buffer. The pH of the solution is adjusted to 10 using 5 M sodium hydroxide (Fig. 3A(i)). The solution is heated to 70℃ in a fume hood, and methacrylic anhydride (MAA) is added at a concentration of 0.0833 mL per gram of gelatin while stirring (Fig. 3A(ii)). After MAA addition, the solution should be covered with aluminum foil and carefully monitored (Fig. 3A(iii)). The reaction proceeds for 2 h with continuous stirring, after which the solution is cooled to room temperature.

#### GelMA purification via precipitation or dialysis

After synthesis, the GelMA product can be purified by either ethanol precipitation or dialysis. Dialysis results in a higher purity than ethanol precipitation but requires a longer preparation time. For dialysis, the GelMA solution is diluted to 5 wt% using ultrapure water, then dialyzed against > 2 L of ultrapure water using a 10 kDa molecular weight cutoff membrane for 5 days at room temperature, exchanging water twice a day for the first 2 days and once a day for the subsequent 3 days. The dialyzed GelMA solution is lyophilized and stored at -80℃ for long-term storage until use.

For precipitation, the GelMA reaction mixture is added to a three-fold volume of ethanol. This process causes the GelMA to precipitate out of the solution, forming a gummy aggregate (Fig. 3A(iv)). If the GelMA mixture appears too fluid, additional fresh ethanol is added to promote full precipitation. The aggregated GelMA is then transferred into a beaker and dried for at least 24 h in the fume hood. After drying, the GelMA aggregate is redissolved with ultrapure water in a 1:1–1:2 weight ratio. The beaker is then placed on a hot plate set to 85–90℃ and heated under constant stirring for at least 1 h to remove residual ethanol, as indicated by the cessation of visible bubbling. Aliquots of the GelMA solution are placed into 50-mL tubes and stored at 4℃ until use (Fig. 3A**(v)**). To determine the concentration of the prepared GelMA, a small amount (e.g. 50–200 mg) of the GelMA solution is pipetted into 3 weigh boats. The wet mass of each aliquot is measured immediately after dispensing. The samples are then left to dry for at least 24 h at room temperature, covered to protect from debris. After drying, the concentration of GelMA is calculated using the following formula: $\:{wt\%\:=\:M}_{dry}\:/\:({M}_{wet}-\:{M}_{dry})\:\times\:\:100\%\:$, where *M*_*wet*_ is the initial wet mass and *M*_*dry*_ is the final dry mass (Fig. 3A(vi)).

The degree of methacrylation strongly dictates the degradation rate, swelling behavior, and stiffness of GelMA hydrogels. Increasing such leads to an increase of methacrylate groups available for crosslinking, forming denser polymer networks and increasing the hydrogel modulus [33, 34]. In the described process, degree of methacrylation can be controlled by adjusting the amount of methacrylic anhydride (MAA) added during the GelMA reaction process.

To determine the degree of methacrylation, the prepared GelMA can be characterized using proton nuclear magnetic resonance (NMR) spectroscopy (Fig. 3B). The aromatic amino acid signal (7.05–7.25 ppm in D2O solvent) was used as the internal reference to normalize the lysine signals of GelMA and gelatin. As methacrylic anhydride reacts with primary amines on lysine residues of gelatin to form GelMA, the degree of methacrylation was calculated as the percentage decrease in the normalized lysine signal in GelMA relative to CWF gelatin:

$$\begin{aligned}&Degree\:of\:methacrylation\cr&=\left(1-\frac{\begin{aligned}&Normalized\:lysine\:integral\cr&\quad\:signal\:of\:GelMA\end{aligned}}{\begin{aligned}&Normalized\:lysine\:integral\cr&\quad\:of\:CWF\:gelatin\end{aligned}}\right)\cr&\times100\%\end{aligned}$$

From this procedure, a degree of methacrylation of 70.7 ± 5.2% was obtained from four separate batches of synthesis.

### 3D printing of perfusable structures

#### Support bath preparation

As a generalizable approach for fabricating perfusable structures, GUIDE-3DP can be applied with a variety of support bath materials. Here, we describe the preparation of support baths using GelMA or alginate as the gel precursor and Aristoflex AVC as a viscosity modifier. In this formulation, the gel precursor enables the printed structure to be crosslinked, while the viscosity modifier enhances the viscosity of the support bath, allowing it to confine printed filaments with high shape fidelity. To encapsulate cells in the support bath, a cell suspension can be mixed with the gel precursor solution, which is then mixed with a viscosity modifier. As an alternative, microparticles consisting of the gel precursor can be used as a standalone support bath that facilitates the spreading of encapsulated cells [35–37].

Prior to support bath preparation, the required volumes of support baths and stock solutions are calculated based on the number of constructs and wells to be used. Typically, approximately 8 g of support bath is used per well in a 6-well plate. Depending on the design, multiple constructs could be printed within the same well, with the understanding that these constructs would be crosslinked at the same time. Excess material should be prepared to account for experimental variability.

**Fig. 4:**
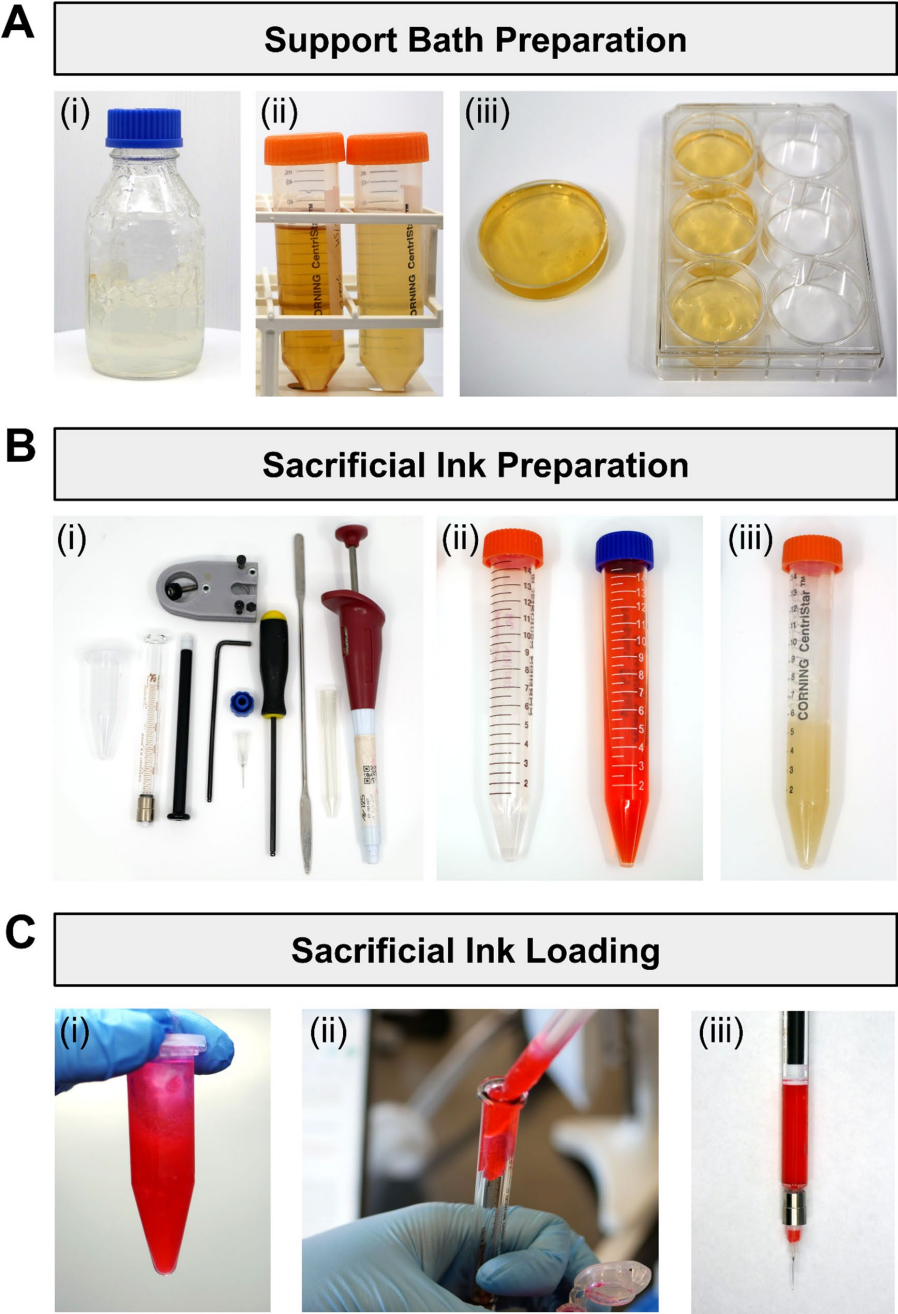
Support bath and sacrificial ink preparation for GUIDE-3DP. **(A)** Support bath preparation: (i) Aristoflex AVC (4 wt%) stock solution, (ii) GelMA (40 wt%) stock solution (left) and GelMA (20 wt%) + AVC (2 wt%) support material (right), and (iii) support baths loaded into dishes for printing. **(B)** Sacrificial ink preparation: (i) supplies needed for ink preparation, (ii) crosslink initiator solutions composed of LAP (50 mM; left) or Eosin Y disodium salt (2 mM; right), and (iii) resuspended gelatin microparticle slurry (100 mg/mL). **(C)** Loading of sacrificial ink into print syringe: (i) as-prepared ink, comprising gelatin microparticles (100 mg/mL), Eosin Y disodium salt (0.1 mM), and pink fluorescent beads, (ii) addition of sacrificial ink to syringe using a positive displacement pipette, and (iii) sacrificial ink loaded into syringe fitted with a 27 G × ½” needle

To prepare the support bath, stock solutions of gel precursor (GelMA or alginate) and viscosity modifier (Aristoflex AVC) are first prepared (Fig. 4A(i) and (ii)). For the gel precursor, a stock solution of GelMA (40 wt%) or alginate (4 wt%) is prepared by diluting the appropriate mass of cold water fish (CWF) GelMA or alginic acid sodium salt in phosphate buffered saline (PBS). The precursor stock solutions are stored at 4℃. For the viscosity modifier, a stock solution of Aristoflex AVC (4 wt%) is prepared by dissolving AVC powder in PBS, taking precaution to avoid powder inhalation by wearing a mask when handling the powder. The AVC powder is allowed to fully dissolve on a magnetic stirrer or shaker at room temperature, and the resultant AVC stock solution is stored at room temperature. Stock solutions are autoclaved on a liquid cycle for sterile use.

To prepare a GelMA support bath, the GelMA stock solution is heated to room temperature, allowing it to liquefy. A support bath composed of 20 wt% GelMA + 2 wt% AVC is prepared by mixing 40 wt% GelMA and 4 wt% AVC stock solutions in a 1:1 ratio. Optionally, 0.5–1 wt% fibrinogen can be added to enhance endothelial cell adhesion. For use of Eosin Y as the crosslink initiator, the co-initiators triethanolamine (TEA) and 1-vinyl-2-pyrrolidinone (NVP) are added at 0.5 wt% and 0.2 wt%, respectively, to the support material. To prepare an alginate support bath, 4 wt% alginate and 4 wt% AVC stock solutions are mixed in a 1:1 ratio.

For preparation of printing dishes, 8 g of support material is dispensed into each required well of a 6-well plate and sealed with Parafilm (Fig. 4A(iii)). Plates are centrifuged at 4,000 rpm for 5 min at room temperature to remove air bubbles and flatten the surface. Alternatively, 50-mL tubes containing the support material are centrifuged at 4,000 rpm for 5 min at room temperature to remove bubbles. The centrifuged support material is poured or dispensed with a positive displacement pipette into an appropriate printing dish. The support bath is stored at 4℃ until use.

#### Sacrificial ink preparation

For ink preparation, all essential supplies are gathered, including print syringes, 27 G × ½” blunt-tip needles, spatulas, 5-mL tubes, a 1-mL positive displacement pipette with capillary tips, and syringe holders (Fig. 4B(i)). For sterile printing, supplies are sterilized by autoclaving or by thoroughly coating with 70% ethanol and drying completely in a tissue culture hood.

For printing using GelMA, crosslink initiator solutions are prepared by dissolving either lithium phenyl-2,4,6-trimethylbenzoylphosphinate (LAP) at 50 mM (14.71 mg/mL) or Eosin Y disodium salt at 2 mM (1.04 mg/mL) in PBS (Fig. 4B(ii)). For printing using alginate, a crosslink initiator solution is prepared by dissolving calcium chloride at 1 M (110.98 mg/mL) in PBS. For sterile printing, the solutions are filtered through a 0.22-µm syringe filter. A sacrificial gelatin microparticle ink (either purchased or prepared as described in [38]) is prepared by resuspending to 100 mg/mL in cold buffer or cell culture medium and gently mixing with a narrow spatula to avoid melting (Fig. 4B(iii)). As an alternative, Pluronic F-127 can be used as the sacrificial ink by dissolving at 26 wt% in cold buffer or cell culture medium. The crosslink initiator is then added to the sacrificial ink at a final concentration of 1–5 mM for LAP, 0.025–0.1 mM for Eosin Y disodium salt, or 50–100 mM for calcium chloride. Optionally, a suspension of fluorescent beads can be included in the ink for visualization. The prepared sacrificial ink is transferred into 2.5-mL Hamilton syringes using a positive displacement pipette, and the syringes are then fitted with 27 G × ½” nozzles for printing (Fig. 4C).

#### (Optional) dual-material nozzle calibration

**Fig. 5:**
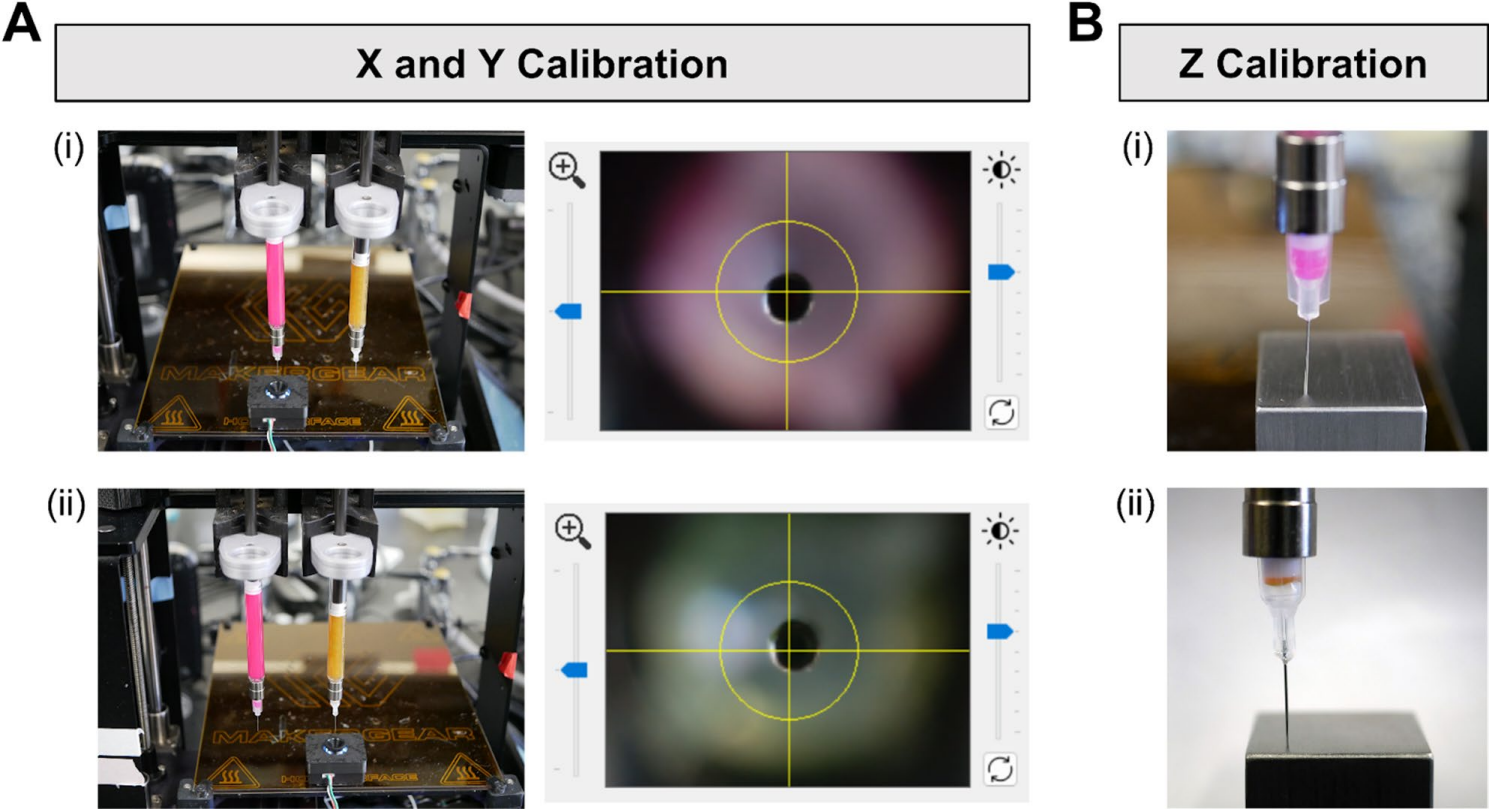
Dual-material calibration process. **(A)** Alignment of print nozzles in the X and Y axes using a calibration camera: (i) Tool 0 and (ii) Tool 1. **(B)** Alignment of print nozzles in the Z axis using a calibration cube: (i) Tool 0 and (ii) Tool 1

To demonstrate the 3D printing of perfusable structures using two sacrificial inks, we utilized a 3D bioprinter with an open-source design comprising two Replistruder syringe pump extruders [39, 40]. The calibration of dual extruders is essential for achieving high-resolution multi-material 3D printing. This process involves precise spatial alignment in the X, Y, and Z axes, typically using a camera-assisted or block-based approach. For the camera-based method, a calibration camera is secured onto the print bed using laboratory tape or double-sided tape. The camera must be positioned such that both needles can reach its field of view. The first tool (i.e. Tool 0) is selected and moved to the center of the camera (Fig. 5A(i)). The Z height is adjusted until the needle is in focus, which typically requires the needle to be a specific distance above the bed. Once positioned, the console commands “G92 × 0”, “G10 P0 × 0”, “G92 Y0”, and “G10 P0 Y0” are used to define Tool 0’s origin and remove any offsets. The second tool (i.e. Tool 1) is then centered on top of the camera, and its displacement relative to Tool 0 is determined using the “M114” command (Fig. 5A(ii)). The offsets for Tool 1 are set using “G10 P1 X(-#)” and “G10 P1 Y(-#)” to reflect its position relative to Tool 0, where # is the corresponding position coordinate from the “M114” command.

Z-axis calibration is performed using a physical reference block placed on the print bed. Tool 0 is lowered until it just touches the surface of the block, which is confirmed by gently tapping the needle tip with forceps; lack of movement indicates proper contact, while movement suggests the tip is not flush (Fig. 5B(i)). The commands “G92 Z0” and “G10 P0 Z0” are used to set Tool 0’s Z origin. Tool 1 is then aligned in the same fashion (Fig. 5B(ii)), and its Z displacement relative to Tool 0 is set using “G10 P1 Z(-#)”, where # is the Z position coordinate from the “M114” command. This procedure should be repeated whenever needles or syringes are swapped to account for any physical variation that might affect precision.

An alternative calibration method involves using a precision-machined reference block to define a known point on the print bed. A reference block is placed at the near right corner of the print bed, and Tool 0 is carefully aligned to the X face of the block using small step movements. The X position of Tool 0 is then set to zero using the “G92 × 0” and “G10 P0 × 0” commands. This alignment process is repeated with the Y and Z faces, with the Y and Z positions being set to zero using corresponding “G92” and “G10” commands. Tool 1 is then aligned sequentially to the X, Y, and Z faces of the same reference block. When the print needle of Tool 1 is brought flush to a given face, the “M114” command is used to determine its position relative to Tool 0, and the coordinates are set using the commands “G10 P1 X(-#)”, “G10 P1 Y(-#)”, and “G10 P1 Z(-#)”, where # is the corresponding coordinate given by the “M114” command.

Once both extruders are calibrated, the print head is moved to the center of the bed, and a new absolute origin is defined with “G92 × 0 Y0 Z0”. The accuracy of calibration can be verified by commanding Tool 0 and Tool 1 to move to the position (X = 0, Y = 0). Tool 1 should move directly to the same center location, validating that both tools are aligned relative to one another. If discrepancies are observed, the offsets can be reset using “G10 P(0 or 1) X(#) Y(#) Z(#)”.

Tool changes between extruders require custom G-code to avoid collisions and ensure proper repositioning. This routine involves lifting the active tool (Tool 0 or 1), selecting the next tool, moving the tool to the desired starting position, and lowering the tool 10 mm. These tool change G-codes can be modified to accommodate specific print setups, such as the height of a support bath or printing dish.

Additional set-up considerations include using interfaces such as Pronterface or Duet Web Control to send G-code and monitor tool positions in real time. For 3D bioprinters with custom-built hardware, components such as the control board and printhead may be upgraded to accommodate dual extruders. G-code sequences for starting and ending a print should be customized accordingly, typically using commands like “G92”, “G1”, and “M302” to define tool positions, reset axes, and allow cold extrusion, respectively. With these procedures, accurate and reproducible dual-material 3D printing can be achieved.

#### Generation of perfusable constructs

**Fig. 6:**
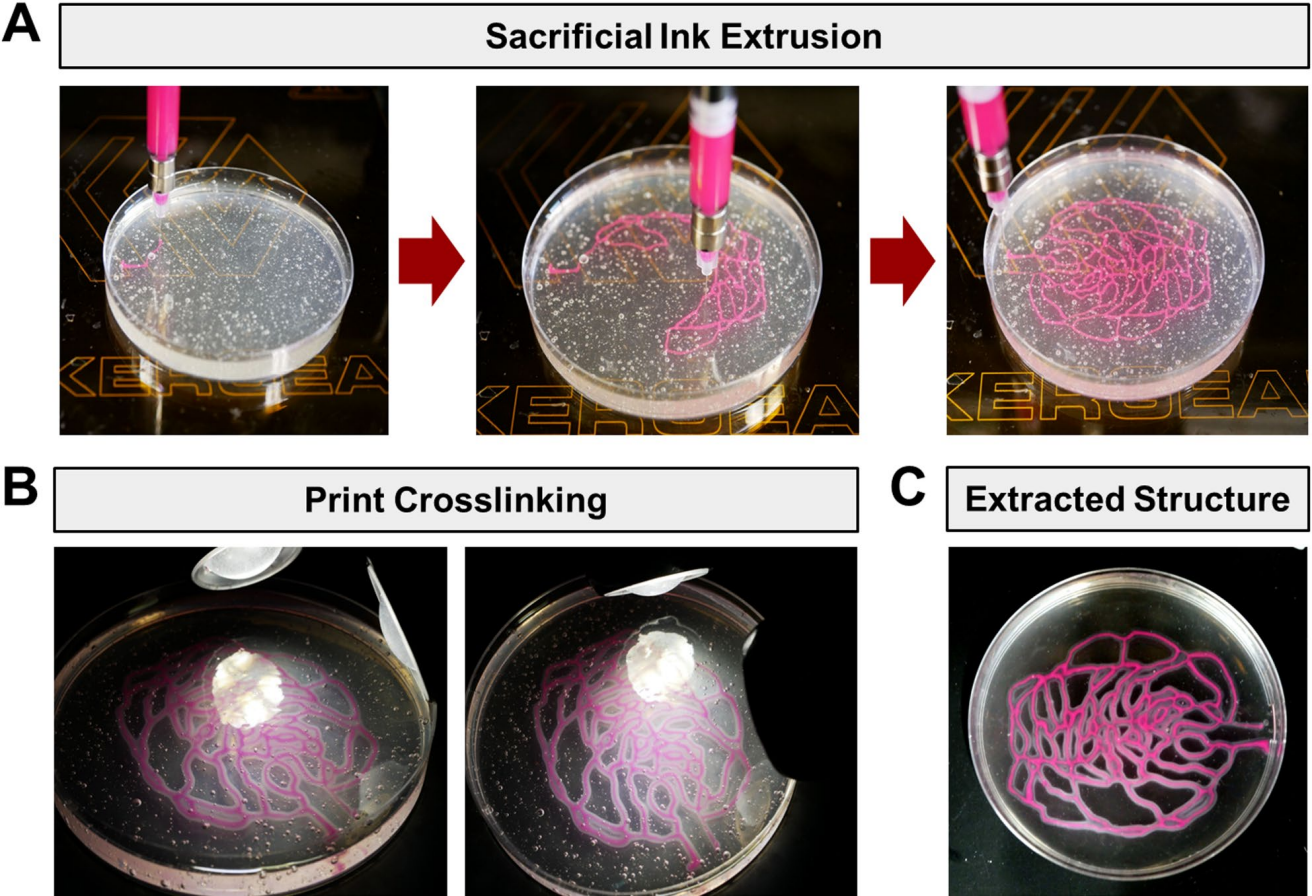
Printing of perfusable GelMA constructs using GUIDE-3DP. **(A)** Photographs of ink extrusion process for a representative retinal vasculature structure (derived from NIH3D [31]). **(B)** Photocrosslinking of printed structure. **(C)** Self-supporting construct obtained after extraction from the support bath

Prior to printing, necessary supplies are collected, including the 3D bioprinter, syringe holders, nozzles, medium for washing, and crosslinking lamp. The G-code file is uploaded to the bioprinter. Support baths are allowed to equilibrate to room temperature. The print syringe is mounted onto the printer and checked for extrusion. Printing is performed by positioning the nozzle within the support bath and executing the G-code file (Fig. 6A). Constructs are incubated at room temperature to allow diffusion of crosslink initiators for a specified time, typically 5–15 min depending on the desired channel wall thickness. For printing using GelMA, photocrosslinking is performed with an ultraviolet (UV) lamp for 5 min when LAP is used as the crosslink initiator. When Eosin Y is used, constructs are crosslinked for 10 min using a white-light microscope illuminator (Fig. 6B). For printing using alginate, crosslinking is terminated after a specified diffusion time by removing the crosslinked structure from the support bath. Following crosslinking, constructs are gently transferred to a new dish and washed using PBS (for GelMA) or HBSS (for alginate) to remove uncrosslinked support material. The samples are then transferred to fresh medium and incubated at 37℃ to melt the sacrificial gelatin microparticle ink. The closed ends of the lumen are excised using a spatula and forceps, and constructs are washed with warm medium to remove residual gelatin (Fig. 6C). If Pluronic is used as the sacrificial ink, the samples are instead incubated at 4℃ and washed with cold medium to remove the sacrificial material.

**Fig. 7:**
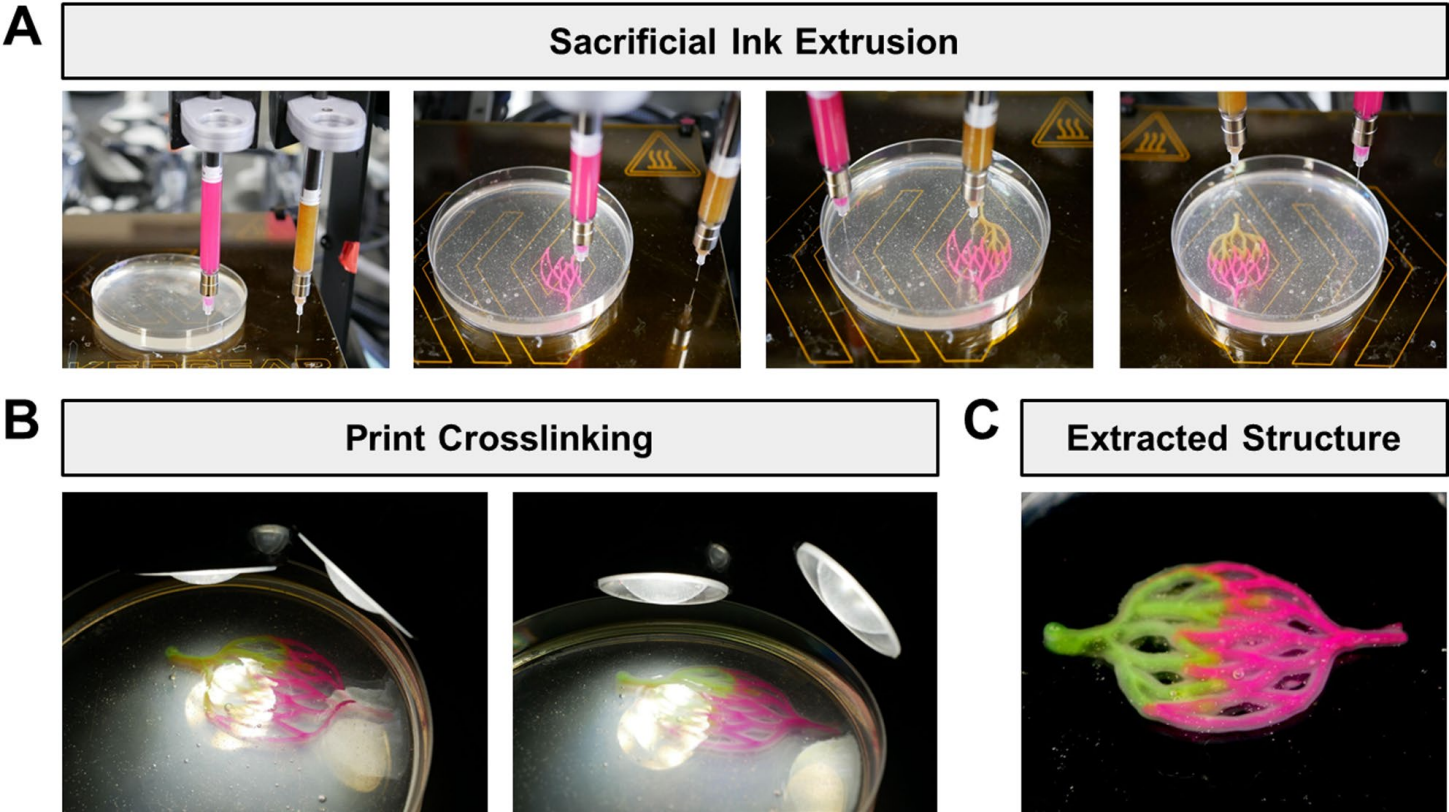
Printing of GelMA construct using two sacrificial inks. **(A)** Photographs of the printing process with two sacrificial inks containing different fluorescent beads. **(B)** Photocrosslinking of dual-material printed structure. **(C)** Self-supporting, dual-material construct after extraction from the support bath

Multi-material printing can be conducted using the same general workflow as single-material printing after performing dual-extruder calibration. The two inks are printed within the same structure (Fig. 7A). After a specified time for crosslink initiator diffusion, the structure is crosslinked via light exposure (Fig. 7B). Extraction of the multi-material structure from the support bath is performed in the same manner as single-material structures (Fig. 7C). While this example demonstrates two inks containing different fluorescent beads, the same strategy can readily be applied to two inks containing different crosslink initiators or cell types.

#### (Optional) endothelialization and culture of printed constructs

**Fig. 8:**
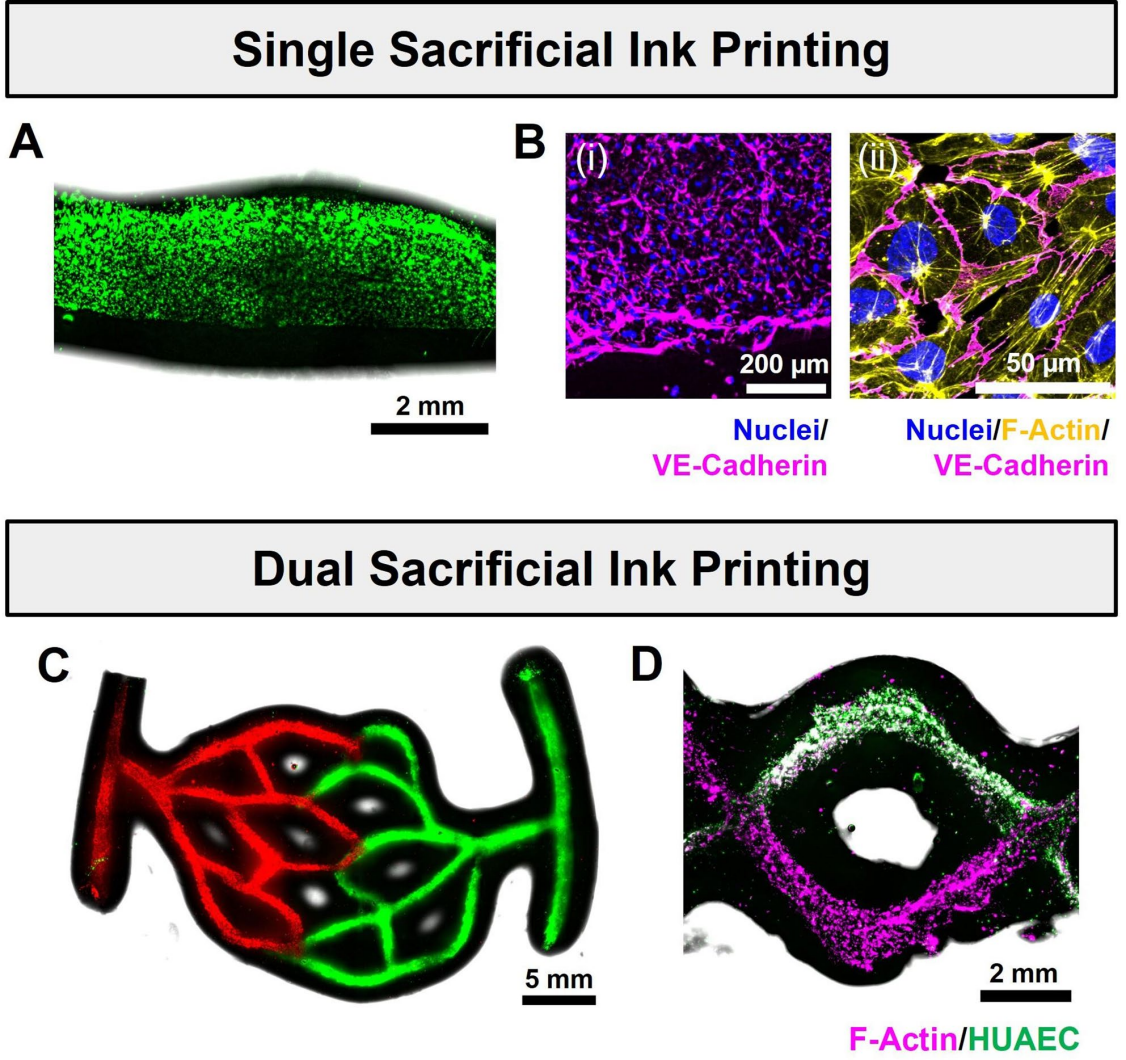
Endothelialization of perfusable constructs via printing of cell-loaded sacrificial inks. **(A)** Channel seeded with human umbilical vein endothelial cells (HUVEC; CellTracker Green-labeled) immediately after sacrificial ink printing. **(B)** Representative immunofluorescence images of HUVEC on printed channel 5 days after sacrificial ink-based endothelialization: (i) Cell distribution at channel wall interface, showing VE-cadherin expression at cell-cell junctions; (ii) magnified view of the endothelial monolayer. **(C)** Vascular-like structure seeded with two different HUVEC populations (RFP- and CellTracker Green-labeled) via printing of two cell-loaded sacrificial inks. **(D)** Intercalating zigzag structure seeded with HUVEC (unlabeled) and human umbilical artery endothelial cells (HUAEC; GFP-labeled) via printing of two cell-loaded sacrificial inks. White regions within the construct indicate overlapping staining of F-actin (which labels the cytoskeleton of both HUVEC and HUAEC), while green regions show HUAEC only. All structures are fabricated with a support bath formulation of 20 wt% GelMA + 0.5 wt% fibrinogen + 2 wt% AVC

To endothelialize printed constructs, cells can be loaded directly within the gelatin microparticle sacrificial ink [30]. Upon gelatin melting, cells included in the ink will be deposited onto the print lumen (Fig. 8A). To enhance cell adhesion, biomolecules such as fibrinogen can be added to the support bath to become entrapped within the perfusable channel walls. Fibrinogen in printed constructs can be crosslinked by incubating in culture medium containing thrombin (5 U·mL^-1^) for at least two hours.

To load the ink with endothelial cells, the pH of the gelatin microparticle suspension is first adjusted to ~ 7 using 1 M sodium hydroxide. A cell suspension is then added to the ink to yield a cell density of 10^7^ mL^-1^. After printing, crosslink initiator diffusion, and crosslinking, the resulting construct is removed from the uncrosslinked support bath, gently washed, and incubated at 37℃ to melt the sacrificial ink, allowing cell sedimentation and adhesion to the lumen surface. After 10 min of incubation, the construct is flipped and incubated for an additional 10 min to allow cell attachment to the opposite luminal surface. The ends of the structure are cut open, and a specified volume of fresh culture medium is added to remove the melted sacrificial ink. This endothelialization strategy can be combined with dual-material bioprinting to spatially pattern two cell types in distinct regions within the same structure (Fig. 8B-C).

Alternatively, cells can be introduced as a separate step after print fabrication by perfusing the lumen with a cell suspension. An endothelial cell suspension with a cell density of 10^7^ mL^-1^ is injected into the print lumen. The construct is incubated for 10 min, then flipped and incubated for 10 min to allow cell attachment to the opposite surface. Finally, a specified volume of fresh culture medium is added to the cell-seeded construct.

To estimate cell retention, the number of suspended cells in the culture medium can be measured using a cell counter. The number of cells retained can then be calculated by subtracting the number of suspended cells from the total number of cells printed or perfused. For structures endothelialized from the sacrificial ink, a high percentage of cells (e.g., 70–90% [30]) is typically retained. This is because cells are confined within the structure during the incubation step at 37℃, during which the sacrificial ink is melted and cells are allowed to adhere to the print lumen.

## General notes and troubleshooting

Table 1 describes issues that may be encountered when designing and fabricating perfusable structures using GUIDE-3DP, along with possible causes and solutions.

**Table 1:** Troubleshooting guide for common problems in GUIDE-3DP

Problem	Possible Reason	Solution
Direction of the print segment is opposite to that expected after exporting coordinates from Rhino3D	Direction of segment was not correctly identified in Rhino3D	Highlight all the desired cells in Excel/Google Sheets, then click Data → Sort Sheet (Z-A). Frequently simulate through G-code editing software to ensure print directions are correct.
Sacrificial ink forms flat sheet rather than circular filament within support bath	Support bath is not viscous enough to support printed structures, or gelatin microparticle ink is melted	A new batch of viscosity modifier, such as AVC stock solution, should be used. Ensure that ink is not melted by avoiding holding the body of the tube or syringe during preparation.
Inhomogeneities or air bubbles in support bath, leading to poor prints	Improper centrifugation and incomplete mixing	Mix vigorously when preparing the support bath. Centrifuge thoroughly to remove air bubbles after mixing.
Ink is too liquid before printing	Holding ink improperly where hands cover most of the tube/syringe, causing it to warm up and melt	Handle tubes/syringes carefully by handling them around the rims and ends so the ink is not in direct apposition to your hands. Make sure the ink is placed in an ice bath throughout preparation.
Ink is not extruded at the start of the print	Ink is not properly primed before initiating the print, or there is clogging of the ink at the nozzle	A macro-extrusion step should be performed before the print is initiated, and/or at the start of the print g-code, to ensure that ink is dispensing correctly.
Structural integrity is weak post-printing	GelMA is partially degraded, crosslink initiator concentration is too low, or light irradiation is insufficient	Increase the concentration of GelMA and/or crosslink initiator to enhance crosslinking. Confirm that the light source can adequately induce crosslinking of the support bath in the presence of a crosslink initiator.

## Expected results

**Fig. 9:**
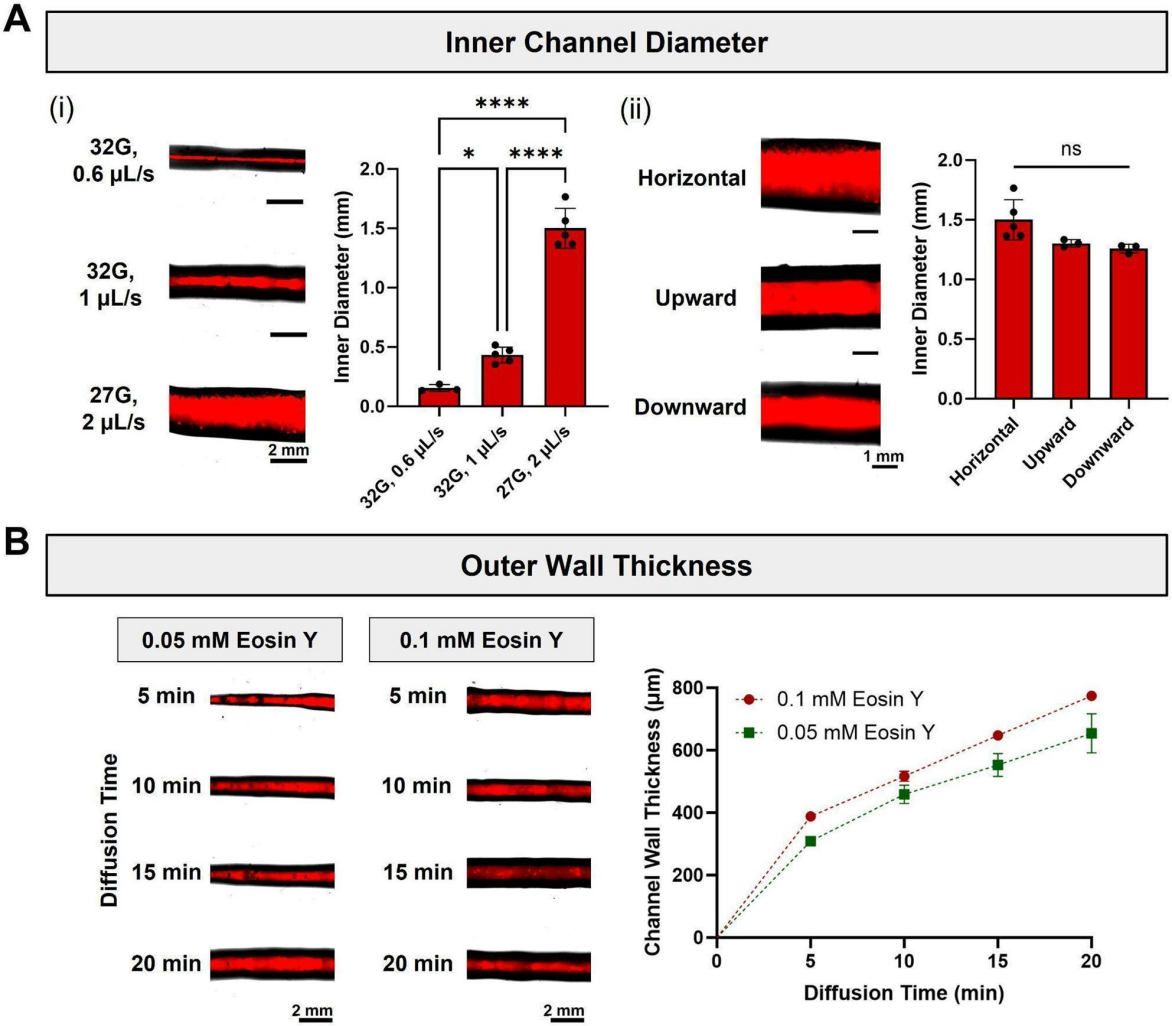
Control over printed channel dimensions in GUIDE-3DP. **(A)** (i) Printed lumen diameter as a function of nozzle size (27 or 32 G) and extrusion rate (0.6, 1, or 2 µL s^-1^), with a fixed printing speed of 180 mm min^-1^. (ii) Printed lumen diameter as a function of extrusion direction, with a fixed extrusion rate of 2 µL s^-1^ and printing speed of 180 mm min^-1^. **(B)** Channel wall thickness as a function of Eosin Y diffusion time, with a fixed extrusion rate of 1 µL s^-1^ and printing speed of 180 mm min^-1^. In all images, the sacrificial ink is labeled with fluorescent beads for visualization

In this protocol, representative vascular-like networks were fabricated to demonstrate GUIDE-3DP’s tunability and versatility, which are necessary features for creating physiologically relevant vascular mimics. Importantly, GUIDE-3DP enables precise and independent control of vessel inner and outer diameters. Firstly, the extrusion rate of the sacrificial ink can be modulated to form varying lumen diameters. For instance, increasing the extrusion rate and/or nozzle size leads to increases in the inner channel diameter (Fig. 9A(i)). This knowledge can be applied to fabricate structures with defined lumen sizes. Apart from extrusion parameters, the printing direction is another notable parameter that must be considered. Printing in the upward, downward, and horizontal directions result in nearly identical lumen sizes, with greater variance observed in the horizontal direction due to filament spreading within the support bath (Fig. 9A(ii)). These data provide insight into how inner diameters will behave along the Y and Z directions, allowing us to leverage this knowledge towards printing freeform 3D structures. In addition, to control the outer channel diameter, the channel wall thickness can be modulated by varying the crosslink initiator concentration and diffusion time (Fig. 9B). At a fixed crosslink initiator concentration, the channel wall thickness increases with initiator diffusion time. Along with this, when comparing the wall thickness at any given time point, a higher initiator concentration results in a greater wall thickness.

## Electronic Supplementary Material

Below is the link to the electronic supplementary material.

Additional File 1

Additional File 2

## Data Availability

All data generated or analysed during this study are included in this published article and its supplementary information files.
